# Impact of Fetuin-A, Lp(a), matrix gla protein and macrophage density on calcific aortic valve disease: a clinical study

**DOI:** 10.1186/s12944-022-01625-x

**Published:** 2022-01-22

**Authors:** Cong Liu, Haifeng Liu, Ting Xie

**Affiliations:** 1 Department of Ultrasound Medicine, Union Hospital, Tongji Medical College, Huazhong University of Science and Technology, 430022 Wuhan, China; 2 Clinical Research Center for Medical Imaging in Hubei Province, 430022 Wuhan, China; 3 Hubei Province Key Laboratory of Molecular Imaging, 430022 Wuhan, China; 4 Department of Medical Engineering, Tongji Medical College, Union Hospital, Huazhong University of Science and Technology, 430022 Wuhan, China; 5 Department of Cardiac Surgery, Hainan General Hospital, No.19 Xiuhua Road, Xiuying District, 571000 Haikou, China

**Keywords:** Fetuin-A, Lp(a), Matrix gla protein, Macrophage density, Calcific aortic valve disease, Clinical study

## Abstract

**Background:**

Calcific aortic valve disease (CAVD) has a substantial and increasing burden in the ageing population with occult onset.Present study aimed to assess association of clinical characteristics of these patients and occurrence of CAVD.

**Methods:**

Patients diagnosed with CAVD and those receiving healthy medical examination in our hospital from January 2019 to February 2021 were enrolled in this retrospective study. Clinical characteristics, ultrasonic indicators, serological indicators and histology of CAVD were collected and compared among different groups. Logistic regression and Pearson correlation analysis was used to explore relationship between these indexes and occurrence of CAVD.

**Results:**

DBP, SBP, LVESD, LVEDD, IVS, PW, AV Vmax, TC, TG, LDL-C, Fetuin-A, Lp(a) in severe group were higher than mild, moderate and control groups (*P*<0.05), while those indexes of patients in moderate group were higher than that in mild and controlled groups (*P*<0.05). Besides, theses indexes of patients in mild group were also higher than that of controlled one (*P*<0.05). However, LVEF, HDL-C and MGP of patients in severe group was the lowest (*P*<0.05), while those in moderate group were lower than mild and controlled groups. Moreover, these indexes in mild group were also lower than control group (*P*<0.05). In Logistic regression analysis, MGP, Fetuin-A and Lp(a) were all independently associated with occurrence of CAVD (*P*<0.05). In Pearson correlation analysis, Fetuin-A and Lp(a) were positively correlated with progression of the disease, while MGP and macrophage density were negatively correlated with it.

**Conclusions:**

Fetuin-A, MPG and Lp(a) were independently associated with the occurrence of CAVD, and they might be potential predictors for diagnosis of this disease.

## Introduction

Calcific aortic valve disease (CAVD) is a common degenerative heart disease with aortic or valve stenosis [1], and it has a substantial and increasing burden in the ageing population with 11% of patients older than 70 years [2]. The degenerative calcified cardiac valve disease is characterized by high disability and mortality, which poses a serious threat to the health of patients. Due to the concealed feature and onset with other senile cardiovascular disease, it is often misdiagnosed.

Fetal globulin-A (Fetuin-A) is a plasma glycoprotein synthesized and secreted by liver cells and leads to osteoblast like differentiation of vascular smooth muscle cells [3]. Lp(a) is a low-density-lipoprotein-like particle which is covalently bound to an apolipoprotein(a)[apo(a)] tail [4]. Matrix Gla protein (MGP) is widely secreted in heart, kidney, arterial wall, bone, lung, skin and other tissues [5]. Macrophages perform important role to the maintenance of tissue homeostasis and tissue repair [6]. A series of studies have founded that Lp(a), Fetuin-A, MGP and macrophage density are correlated with incidence and prognosis of CAVD [5, 7–10]. However, limited reports exist regarding Fetuin-A, Lp(a), MGP and macrophage density among elderly patients.

The present study aimed to assess the association between clinical characteristics, including Fetuin-A, Lp(a), MGP and macrophage density, and occurrence of CAVD on old patients.

## Methods

### Participants

1. Patients harboring CAVD and patients receiving healthy medical examination in Union hospital affiliated to Tongji Medical College from January 2019 to February 2021 were enrolled in this retrospective study. Patients meeting these *criteria* were included: (I) patients diagnosed with heart valve disease according to the American Heart Association/American College of Cardiology (AHA/ACC) 2014 guideline [11]; (II) indication of the aortic valve calcification based on echocardiography; (III) valve thickness exceeds 3 mm or more, with hard valve leaflet and enhanced echo; (IV) patients signed the informed consents. Patients were excluded according to criteria below: (I) abnormal chronic renal, liver, kidney function or combined with malignancy; (II) congenital cardiac valve disease, rheumatic heart valve disease, pulmonary heart disease, cardiomyopathy, valve disease caused by infective endocarditis; (III) abnormal immune system, hyperthyroidism, anemia; (IV) non-steroidal anti-inflammatory drug and hormones within 6 months; (V) history of chronic inflammatory; (VI) systemic disease, including parathyroid disease and renal failure that may interfere calcium phosphorus metabolism.

Patients with CAVD were allocated to 3 groups according to the aortic valves (AV) pressure in the echocardiography as follow: mild: the maximum pressure differential and the average pressure step difference were 5 to 30mmHg and 4 to 20mmHg, respectively; moderate: the maximum pressure differential and the average pressure step difference were 30 to 60mmHg and 20 to 50mmHg, respectively; severe: the maximum pressure differential and the average pressure step difference were > 60mmHg and >50mmHg, respectively. Patients receiving healthy medical examination were designed as control.

The research program was in line with requirements of the World Medical Association Helsinki Declaration. The study was approved by ethical committee of Union Hospital, Tongji Medical College, Huazhong University of Science and Technology with number of UHEIC-20-D19.

### Data collection

General information: age, gender, body mass index (BMI), smoking history, drinking history, hypertension history, diastolic blood pressure (DBP), systolic blood pressure (SBP), etc.

Echocardiography: all participants were tested by chest two-dimension echocardiogram using Philips EPIQ7c. Valve ring and root of valve were thickened >3mm, and leaflet activity was limited by calcification of aortic valve. All patients with CAVD underwent valve replacement. In the view of the long axis next to the sternum bone, left ventricular end systolic dimension (LVESD), left ventricular end diastolic dimension (LVEDD), left ventricular ejection fraction (LVEF), interventricular septum thickness (IVS), left ventricle posterior wall (PW) thickness were measured.

Serological indicators: 8ml venous blood were taken in the morning after admission to hospital and divided into two parts: one part was centrifuged at 3500r/min for 5 min at room temperature to separate serum, and stored at -20℃. Then it was taken to measure total cholesterol (TC), triglyceride (TG), low density lipoprotein cholesterol (LDL-C) and high density lipoprotein cholesterol (HDL-C) by enzyme colorimetry methods. Another part was taken to measure Fetuin-A and MGP by enzyme-linked immunosorbent assay. Lp(a) was also determined in this part by immunoturbidimetry.

Hematoxylin-eosin (HE) staining: HE staining were routinely performed for all specimens, and valve thickness, tissue structure, cell composition, calcified site and changes around calcification were observed under microscope.

Immunohistochemical staining: rabbit anti human CD68 monoclonal antibody was purchased from Wuhan BOSTER Biological Technology Co., Ltd. It was diluted at 1:100 and with the use of pv-9000 general two-step kit. It was considered positive if it was brownish yellow and higher than the background color. According to the semi quantitative evaluation of positive cell counting, 4 high-power visual fields were selected, and divided into 4 grades by the proportion of positive cells. Grade 0: < 10% cells show positive immune reaction; Grade 1: < 25% of immune positive cells; Grade 2: 25% ~ 50% of the cells were positive; Grade 3: > 50% of cells showed positive immune reaction.

Image analysis of immunohistochemical staining: CD68 positive cells were measured by IPP (Image Pro Plus) software, and the correlation between the maximum cell density and the clinical data were analyzed.

Observation indexes: ultrasonic and serological indexes of each group were compared. The visual observation, HE staining and CD68 immunohistochemical staining results of valve specimens were collected. Correlations between these indexes and occurrence of degenerative calcified aortic valve disease were explored.

### Statistical analysis

SPSS 21.0 software was used to analyze the data, and excel was used to establish the database. Normally distributed measurement data were expressed as mean±standard deviation (SD), and the comparisons were examined by one-way ANOVA. The categorical data were expressed as n(%), and the differences between the two groups were examined by chi-square analysis. Unconditional binary logistic regression and Pearson correlation analysis were used to explore correlations between observation indexes and occurrence of degenerative calcified aortic valve disease. Odds ratio (OR) and 95% confidence interval (CI) were generated to present the effect of risk factors for predicting CAVD. A *P*-value below 0.05 was considered statistical difference.

## Results

### Baseline characteristics

Totally 180 patients harboring CAVD and 182 patients receiving healthy medical examination enrolled in this analysis. There were 39 males and 30 females in the mild group, 61 to 85 years old, with an average age of (69.76 ± 8.36). There were 33 males and 25 females in the moderate group, 61 to 84 years old, with an average age of (69.69±8.32). Besides, there were 31 males and 22 females in severe group, 61 to 85 years old, with an average age of (69.72±8.45). Moreover, there were 105 males and 77 females in controlled group, 61 to 84 years old, with an average age of (69.68 ± 8.27). The general data were comparable among these 4 groups (*P*>0.05) (Table 1).

**Table 1:** Comparison of the general information of each group

Item	Mild group (*n*=69)	Moderate group (*n*=58)	Severe group (*n*=53)	Control group (*n*=182)	Z/F	*P*
Gender (male/female)(n, %)	39/30	33/25	31/22	105/77	0.059	0.996
Age (years)	69.76 ± 8.36	69.69±8.32	69.72±8.45	69.68 ± 8.27	0.388	0.762
BMI (kg/m^2^)	25.71±2.38	25.65±2.34	25.59±2.37	25.41±2.42	0.342	0.795
Smoking history (n)	26	27	22	78	1.069	0.785
Drinking history (n)	25	23	21	62	0.928	0.818
Hypertension history (n)	30	26	26	76	0.928	0.819
DBP(mmHg)	128.9±12.1^@^	130.8±11.1^*@^	133.0±12.0^#*@^	126.3±11.2	5.746	0.001
SBP(mmHg)	89.1±9.9^@^	91.9±9.4^*@^	93.3±9.4^#*@^	88.0±9.0	632.065	<0.001

Note: compared with mild groups ^#^*P*<0.05; compared with moderate group ^*^P<0.05; compared with the control group ^@^*P*<0.05.

Abbreviation: BMI: body mass index; DBP: diastolic blood pressure; SBP: systolic blood pressure

General data, including age, gender, BMI, smoking history, drinking history, and hypertension history, were not significantly different between any two of these 4 groups (*P*>0.05), respectively. DBP and SBP of patients in the severe group were higher than that of mild, moderate and controlled groups. while these of moderate group were higher than that of mild (*P*<0.05). Besides, DBP and SBP of patients in mild group were also higher than that of controlled one (*P*<0.05) (Table 1).

### Ultrasonic and serological indicators

LVESD, LVEDD, IVS, PW, AV Vmax, TC, TG, LDL-C, Fetuin-A, Lp(a) of patients in severe group were higher than that of mild, moderate and controlled groups. Those indexes mentioned above of patients in moderate group were higher than that of mild and controlled groups, while those of mild group were higher than that of controlled one, with significant difference (*P*<0.05). In contrast, LVEF, HDL-C and MGP of patients in severe group were the lowest, followed by moderate, mild and controlled group. The differences between any two of these 4 groups were significant (*P*<0.05) (Table 2).

**Table 2:** Comparison of ultrasonic indicators and serological indicators

Item	Mild group (*n*=69)	Moderate group (*n*=58)	Severe group (*n*=53)	Control group (*n*=182)	Z/F	P
LVEF(%)	58.32±5.21	56.03±5.32	53.21±5.28	60.87±5.76	31.288	<0.001
LVESD(mm)	30.04±3.19	33.76±3.21	35.82±3.48	26.87±2.98	149.111	<0.001
LVEDD(mm)	43.98±4.21	46.92±4.34	50.76±4.87	38.78±4.87	112.831	<0.001
IVS(mm)	8.78±1.23	9.93±1.31	12.98±1.26	6.98±1.08	385.553	<0.001
PW(mm)	9.87±0.98	10.98±0.92	12.97±1.07	7.87±1.02	415.458	<0.001
AV Vmax(m/s)	2.27±0.36	3.87±0.41	4.76±0.39	1.26±0.42	1348.648	<0.001
TC(mmol/L)	4.37±0.36	4.58±0.41	4.78±0.34	4.12±0.33	60.783	<0.001
TG(mmol/L)	1.78±0.29	1.91±0.34	2.08±0.27	1.56±0.31	49.161	<0.001
LDL-C(mmol/L)	2.43±0.23	2.62±0.27	2.87±0.23	2.23±0.23	116.627	<0.001
HDL-C(mmol/L)	1.61±0.23	1.51±0.21	1.38±0.32	1.78±0.33	31.341	<0.001
Fetuin-A(mg/mL)	0.102±0.026	0.122±0.021	0.145±0.025	0.073±0.018	176.232	<0.001
Matrix Gla protein(ng/mL)	7.01±0.37	3.45±0.34	2.87±0.31	11.76±0.36	7.192	<0.001
Lp(a)(mg/L)	265.98±22.91	298.87±23.98	360.91±23.21	196.39±21.87	869.216	<0.001

Abbreviation: LVEF: left ventricular ejection fraction; LVESD: left ventricular end systolic dimension; LVEDD: left ventricular end diastolic dimension; IVS: interventricular septum thickness; PW: posterior wall; AV: Aortic valve velocity; TC: total cholesterol; TG: triglyceride; LDL-C: low density lipoprotein cholesterol; HDL-C: high density lipoprotein cholesterol

### Valve visual observation and HE staining

**Fig. 1:**
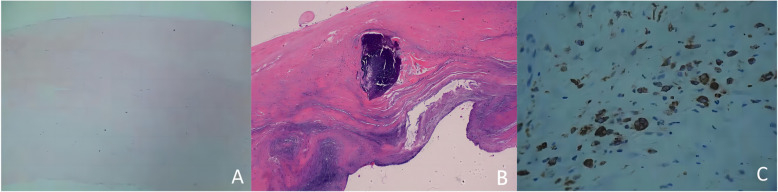
Results of valve visual observation by HE staining and CD68 immunohistochemical staining; (**A)**: normal aortic valve (HE, × 40), composed of collagen layers, loose layers, and ventricular layers, collagen layer (mainly composed of collagen), intermediate loose layer (containing a small amount of collagen), and ventricular layer (mainly composed of elastic fibers); (**B)**: Calcific aortic valve (HE, × 40), visible calcification; (**C)**: Calcific aortic valve (IHC, × 100), CD68 positive macrophage

The normal valve is mainly composed of 5 layers: the endothelial layer on the aortic side, collagen layer rich in collagen fibers, loose layer with less dense matrix and arrangement, ventricle layer with obvious elastic fibers and endothelial layer layer with significant elastic fibers. Normal valve fibers arranged neatly, without blood vessels or inflammatory cell infiltration (Fig. 1 A), while degenerative valve was thicker than normal one. The nodule calcification was presence in the valve loose layer, and the fibrous tissue around it lost its original structure, with formation of new blood vessel, foam cells and adipocytes (Fig. 1B).

### CD68 immunohistochemical staining

CD68 is expressed in the cell membrane. CD68 negative or grade 1 were expressed in the normal valve, and was mainly negatively expressed (85.7%). However, CD68 was primarily expressed up to grade 2 to 3 in the specimen with degenerative valve calcification (Fig. 1 C), which was significant (*P*<0.05). Lipid cells such as macrophage cells and fat cells were mainly located in calcification area (Table 2).

### Risk factors of degenerative calcified aortic valve disease

Based on unconditional binary Logistic regression analysis, MGP (OR: 1.021, 95%CI: 0.062-1.334, *P*<0.001) and Fetuin-A (OR: 0.209, 95%CI: 0.101-0.442, *P*<0.001) were both protective factors for degenerative calcified aortic valve disease, while the adverse effect of Lp(a) (OR: 5.798, 95%CI: 3.001-8.678, *P*=0.003) (Table 3).

**Table 3:** Unconditional binary Logistic regression analysis of influencing factors on elderly degenerative CAVD

Variables	β value	SE value	Wald value	OR value (95%CI)	*P*-value
LVESD	0.587	0.859	0.467	0.728 (0.509-2.765)	0.084
LVEDD	0.312	0.265	6.209	0.809 (0.608-2.098)	0.102
IVS	0.567	0.354	2.675	1.809 (0.887-3.287)	0.093
PW	0.133	0.359	0.128	0.873 (0.427-1.676)	0.709
AV Vmax	0.219	0.378	0.365	1.176 (0.601-2.321)	0.576
DBP	0.528	0.357	2.157	0.578 (0.267-1.189)	0.139
SBP	0.576	0.359	2.581	0.553 (0.269-1.138)	0.103
LVEF	0.591	0.359	2.751	1.813 (0.891-3.287)	0.093
HDL-C	0.211	0.354	0.347	1.221 (0.609-2.512)	0.547
Matrix Gla protein	3.043	0.502	35.765	1.021 (0.062-1.334)	<0.001
TC	0.587	0.354	2.765	0.538 (0.256-1.102)	0.087
TG	0.378	0.356	1.178	0.668 (0.321-1.368)	0.269
LDL-C	0.621	0.346	3.072	0.521 (0.259-1.069)	0.071
Fetuin-A	1.529	0.368	16.676	0.209 (0.101-0.442)	<0.001
Lp(a)	1.642	0.219	70.465	5.798 (3.001-8.678)	<0.001

Abbreviation: LVEF: left ventricular ejection fraction; LVESD: left ventricular end systolic dimension; LVEDD: left ventricular end diastolic dimension; IVS: interventricular septum thickness; PW: posterior wall; AV: Aortic valve velocity; TC: total cholesterol; TG: triglyceride; LDL-C: low density lipoprotein cholesterol; HDL-C: high density lipoprotein cholesterol; DBP: diastolic blood pressure; SBP: systolic blood pressure; SE: standard error; OR: odds ratio; CI: confidence interval

### Correlation of MGP, Fetuin-A, Lp(a) and macrophage density to CAVD

**Fig. 2:**
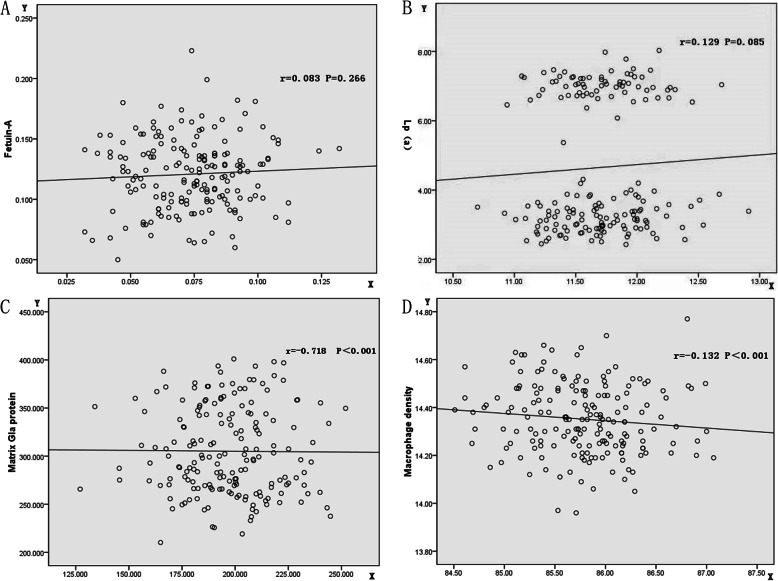
Correlation between variables (Fetuin-A, Lp(a), MGP and macrophage density) and occurrence of CAVD. (**A)**: Correlation between Fetuin-A and CAVD; (**B)**: Correlation between Lp(a) and CAVD; (**C)**: Correlation between MGP and CAVD; (**D)**: Correlation between macrophage density and CAVD; CAVD: calcific aortic valve disease

Based on the Pearson correlation analysis, Fetuin-A and Lp(a) were positively correlated with severity of degenerative calcified aortic valve disease (r=0.083 and 0.129; *P*=0.266 and -0.085, respectively), while MGP and macrophage density were negatively correlated with it (r= -0.718 and -0.132, respectively; both *P*<0.001) (Fig. 2).

## Discussion

CAVD is caused by degenerative pathogenesis and calcium precipitation increasing with age [12]. Due to the occult onset and slow progression of disease, there may be no obvious symptom for decades. With the exacerbation of disease, it can lead to fainting and sudden death. Thus, it is of vital importance to better understand risk factors of valve calcification. Present study showed that patients with high Fetuin-A, high Lp(a) and low MGP should seek early examination and diagnosis for CAVD. A study conducted by Kocyigit et al. showed that compared with patients without valve calcification, the level of serum Fetuin-A in patients with aortic valve calcification was significantly higher [13]. Di Minno et al. analyzed 2283 patients with aortic stenosis and 1549 healthy people. The results showed that compared with the healthy control group, the serum Fetuin-A level in the calcification group increased significantly [14]. It was confirmed that Fetuin-A can prevent aortic membrane calcification by inhibiting ectopic calcification [15]. Lp(a) is recognized as cardiovascular risk factor and closely related to coronary heart disease [16]. Capoulade et al. conducted a study enrolling 220 patients with aortic stenosis for an average follow-up time of 3.5 years, and found that the Lp(a) increased with the worsen progression of disease, which also increased risk of aortic valve replacement and cardiac death [17]. A study conducted by Arsenault et al. showed that Lp(a) was a risk factor for CAVD [18]. As to the impact of MGP, Capoulade et al. proposed that the level of MGP was correlated with aortic valve calcification [19]. Mant et al. proposed that serum MGP was closely related to the risk of coronary heart disease, and lower in patients with atherosclerosis than normal ones [20].

Macrophages are an important for innate immunity and play a central role in inflammation and host defense. Monocyte macrophages are usually described as a type of cells full of diversity and plasticity, mainly because they can generate corresponding adjustments with the changes of the surrounding environment. Otto et al. found that a large amount of macrophages infiltrated under the endothelium in patients with degenerative calcified aortic valve disease after autopsy [21]. In this study, the valves of degenerative diseases were thicker than normal valves. Nodule calcification was located at the loose layer of valve, and the surrounding fibrous tissue were with destructed structure, foam cells and adipocytes.

## Study strengths and limitation

Present case-control study enrolled a number of patients diagnosed with CAVD in a single center, which showed the association between clinical characteristics and occurrence of this disease. It enhanced some evidences discovered already and added the references. There were also several limiations in this study. Firstly, there was inherent biases in this study due to its retrospective nature, more prospective study should be conducted in the future. Secondly, small sample size of present study resulted in restricted strength this analysis. More cohort studies with large sample were still wanted. Thus, conclusions of this study should be interpreted cautiously.

## Conclusions

In conclusion, Fetuin-A and Lp(a) were more highly secreted in the CAVD, while the MPG was poorly secreted with the progression of disease. All these 3 factors were independently associated with the occurrence of CAVD, and they might be potential predictors for diagnosis of this disease.

## Data Availability

The datasets generated and analyzed during the current study are available from the corresponding author on reasonable request.

## Abbreviations

CAVD: Calcific aortic valve disease
Fetuin-A: Fetal globulin-A
MGP: matrix Gla protein
BMI: body mass index
DBP: diastolic pressure
SBP: systolic pressure
TC: total cholesterol
TG: triglyceride
LDL-C: low density lipoprotein cholesterol
HDL-C: high density lipoprotein cholesterol
AV: aortic valves
LVESD: left ventricular end systolic dimension
LVEDD: left ventricular end diastolic dimension
LVEF: left ventricular ejection fraction
IVS: interventricular septum thickness
PW: left ventricle posterior wall
HE: hematoxylin-eosin
SD: standard deviation
OR: odds ratio
CI: confidence interval
