# Correction: Impact of Fetuin-A, Lp(a), matrix gla protein and macrophage density on calcific aortic valve disease: a clinical study

**DOI:** 10.1186/s12944-023-01816-0

**Published:** 2023-04-28

**Authors:** Cong Liu, Haifeng Liu, Ting Xie

**Affiliations:** 1 Department of Ultrasound Medicine, Union Hospital, Tongji Medical College, Huazhong University of Science and Technology, Wuhan, 430022 China; 2 Clinical Research Center for Medical Imaging in Hubei Province, Wuhan, 430022 China; 3 Hubei Province Key Laboratory of Molecular Imaging, Wuhan, 430022 China; 4 Department of Medical Engineering, Tongji Medical College, Union Hospital, Huazhong University of Science and Technology, Wuhan, 430022 China; 5 Department of Cardiac Surgery, Hainan General Hospital, No.19 Xiuhua Road, Xiuying District, Haikou, 571000 China

**Correction: Lipids in Health and Disease 21, 14 (2022)**

**https://doi.org/10.1186/s12944-022-01625-x**

Following publication of the original article [1], the author requested to replace Fig. 1. The correct figure is given below.

**Fig. 1:**
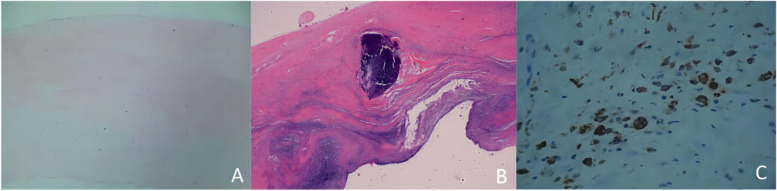
Results of valve visual observation by HE staining and CD68 immunohistochemical staining; (**A**): normal aortic valve (HE, × 40), composed of collagen layers, loose layers, and ventricular layers, collagen layer (mainly composed of collagen), intermediate loose layer (containing a small amount of collagen), and ventricular layer (mainly composed of elastic fibers); (**B**): Calcific aortic valve (HE, × 40), visible calcification; (**C**): Calcific aortic valve (IHC, × 100), CD68 positive macrophage

The original article [1] has been updated.

## References

[1] Liu C, Liu H, Xie T (2022). Impact of Fetuin-A, Lp(a), matrix gla protein and macrophage density on calcific aortic valve disease: a clinical study. Lipids Health Dis.

